# Antiferromagnetic interlayer exchange coupled Co_68_B_32_/Ir/Pt multilayers

**DOI:** 10.1038/s41598-023-49976-4

**Published:** 2024-01-02

**Authors:** Emily Darwin, Riccardo Tomasello, Philippa M. Shepley, Nathan Satchell, Mario Carpentieri, Giovanni Finocchio, B. J. Hickey

**Affiliations:** 1 School of Physics and Astronomy, University of Leeds, Leeds, LS2 9JT UK; 2 Department of Electrical and Information Engineering, Politecnico Di Bari, Via E. Orabona 4, 70125 Bari, Italy; 3 Department of Mathematical and Computer Sciences, Physical Sciences and Earth Sciences, University of Messina, 98166 Messina, Italy; 4 Present Address: Department of Physics, Texas State University, San Marcos, TX 78666 USA

**Keywords:** Magnetic properties and materials, Spintronics

## Abstract

Synthetic antiferromagnetic structures can exhibit the advantages of high velocity similarly to antiferromagnets with the additional benefit of being imaged and read-out through techniques applied to ferromagnets. Here, we explore the potential and limits of synthetic antiferromagnets to uncover ways to harness their valuable properties for applications. Two synthetic antiferromagnetic systems have been engineered and systematically investigated to provide an informed basis for creating devices with maximum potential for data storage, logic devices, and skyrmion racetrack memories. The two systems considered are (*system 1*) CoB/Ir/Pt of N repetitions with Ir inducing the negative coupling between the ferromagnetic layers and (*system 2*) two ferromagnetically coupled multilayers of CoB/Ir/Pt, coupled together antiferromagnetically with an Ir layer. From the hysteresis, it is found that system 1 shows stable antiferromagnetic interlayer exchange coupling between each magnetic layer up to N = 7. Using Kerr imaging, the two ferromagnetic multilayers in system 2 are shown to undergo separate maze-like switches during hysteresis. Both systems are also studied as a function of temperature and show different behaviors. Micromagnetic simulations predict that in both systems the skyrmion Hall angle is suppressed with the skyrmion velocity five times higher in system 1 than system 2.

## Introduction

Synthetic antiferromagnets (SAFs) are an attractive and advantageous material platform, promising to improve the performance of spintronic devices^1^. When placing a non-magnetic spacer between two ferromagnetic materials, the ferromagnetic layers can experience interlayer exchange coupling (IEC)^2–4^. Particular spacers, such as Ir and Ru, cause either ferromagnetic or antiferromagnetic (AFM) coupling depending on their thickness, via the Ruderman–Kittel–Kasuya–Yoshida (RKKY) interaction^5–8^. The AFM coupling promotes the formation of a SAF.

SAFs are appealing for device applications based on magnetic recording, spin orbit torque magnetoresistive random access memory (SOT-MRAM) and spin-transfer torque MRAM^9–11^. More specifically, SAFs in which the ferromagnetic layers exhibit perpendicular magnetic anisotropy (PMA) are also a current focus for soliton applications^12,13^, including skyrmion applications^14,15^, as the opposite spin orientation of consecutive magnetic layers can suppress the skyrmion Hall effect (SkHE)^16–20^. Recent studies have experimentally stabilized skyrmions in SAFs^21–23^, and shown skyrmion bubbles in SAFs being driven with smaller current densities than their ferromagnetic counterparts and with a negligible SkHE^24^. As well as current-induced motion devices, SAFs could be exploited for ultrafast frequency devices^25–28^, similarly to bulk AFM materials. However, an advantage of SAFs over AFM materials is the possibility to study them easily by conventional techniques used for ferromagnets^12^.

A growing number of studies have been focusing on SAF properties, such as tuning the IEC^29^ and Dzyaloshinskii–Moriya interaction (DMI)^30^, SOT switching^31^, annealing effects on the SAF structure^32^, and analyzing the role of the PMA^32,33^. Nonetheless, there is still room to design novel SAF systems to promote more efficient magnetization dynamics. First, the ferromagnetic material used in previous studies is predominantly Co^29,31–34^, however, SAFs can be created with different material combinations. Second, the role of temperature on SAF systems^4,35^ has been studied only for a limited range of materials^36–40^, leading to the following conclusions: (1) the IEC constant decreases with temperature, (2) the SAF state is stable to low temperatures, below 100 K. Third, the effect of spacer layer variations on the SAF properties is usually investigated, while the effect of changes of the ferromagnetic material composition and thickness has received little attention.

Here, we advance the knowledge of SAF systems and test the stability of the SAF against an increasing number of repetitions, against different temperatures, and test the limits of the SAF when coupling together different amounts of magnetic material. We present a SAF material platform which unlocks a realm of possibilities owed to the fine tuning of the thickness of CoB, Ir and Pt allowing the RKKY interaction, interfacial DMI and PMA to be controlled. Amorphous CoB has lower pinning than Co or annealed CoFeB, making it favorable for applications that use domain walls or skyrmions as information carriers^41^. Moreover, the amorphicity promotes a device that is more robust against oxidation effects^42^. The Pt and Ir situated either side of the CoB not only provide an additive DMI^43^, but also extra degrees of freedom to manipulate the IEC.

We analyze two systems: (*System 1*) CoB/Ir/Pt of N repetitions ([CoB/Ir/Pt]_xN_) with Ir inducing the negative coupling between the ferromagnetic layers, and (*System 2*) two ferromagnetically coupled multilayers of CoB/Ir/Pt, coupled together antiferromagnetically with an Ir layer^23,44,45^. We consider the effect of N on the AFM-IEC and observe individual switching for each CoB layer up to seven repetitions. Unlike Ref.^30^, which considers only certain N values, we consider repetitions from two to eight and we observe well defined switches, rather than loop crossing. System 2 is designed to be uncompensated to allow the magnetic textures to be observed more easily with various standard imaging techniques, such as Kerr microscopy, due to a larger read out signal. Similar device designs have been investigated by coupling together only Co/Pt multilayers^44,46^, or Co/Pd for skyrmion investigations^23^.

In addition, we systematically study the IEC in the two systems as a function of temperature, a parameter that is critical when considering material for device implementation and observe distinct behaviors. System 1 maintains the SAF phase down to 10 K^36,47^, whereas, system 2 shows a phase change to a more ferromagnetic-type state below 250 K.

Finally, we perform micromagnetic simulations to demonstrate the potentiality of the systems for skyrmion-based devices. We show the suppression of the skyrmion Hall angle and a velocity-current relation for system 1 five times higher than system 2.

Therefore, our results not only shed more light on the properties of SAF devices, but also open the way to future developments of SAF high performance racetrack memories based on skyrmions and domain walls.

## Results and discussion

Two SAF systems grown via magnetron sputtering (see “Methods” section) are probed for this work. When engineering these systems, it is crucial to extract the thickness of the materials, especially the Ir thickness required to induce the AFM coupling. These thicknesses were computed by doing low angle X-ray reflectometry scans, and fitting them with GenX^48^ (see Supplementary Fig. S1a and b).

We will first discuss both systems separately, then compare their properties.

### System 1: [CoB/Ir/Pt]_xN_

**Figure 1:**
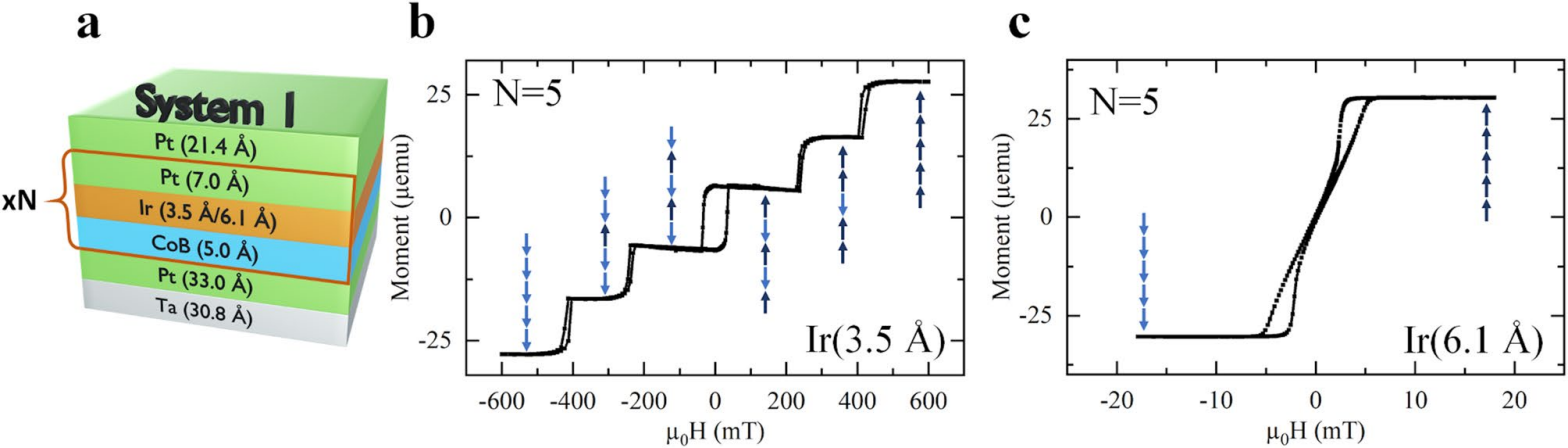
Sketch of system 1 and corresponding hysteresis measurements (**a**) The repetition of CoB/Ir/Pt N times with the Ir thickness causing all the CoB layers to couple together antiferromagnetically or ferromagnetically. There is also a base of Ta and Pt, and a Pt capping layer. (**b**,**c**) Hysteresis loop performed using SQUID magnetometry with a magnetic field applied out-of-plane. The arrows represent the spin orientations of the five layers, light blue down arrows and dark blue up arrows. (**b**) The loop of system 1 with five repetitions and an Ir thickness of 3.5 Å. There is an individual switch for each CoB layer indicating the AFM-IEC between all the ferromagnetic layers. Here, the arrows represent an example switching mechanism. (**c**) The hysteresis loop of system 1 with five repetitions and an Ir thickness of 6.1 Å. This hysteresis loop shows that all the CoB layers switch together via ferromagnetic coupling. This measurement was corrected for an offset in the x-axis due to trapped flux.

Figure 1a shows the system referred to as system 1, designed with N repetitions of CoB with a spacer of Ir and Pt. The Ir mediates a negative IEC between adjacent CoB layers. From hysteresis loops measured with a superconducting quantum interference device (SQUID) and the magneto-optical Kerr effect (MOKE), we find that the AFM coupling peaks occur at Ir thicknesses of 3.5–5 Å, and 12.5–14 Å (see Supplementary Fig. S1c), in agreement with results found in Refs.^8,30^. Supplementary Fig. S1d and e show the coupling strength as a function of Pt and CoB thickness.

SQUID hysteresis loops of system 1 with five repetitions, grown with two different thicknesses of the Ir layer, 3.5 Å (Fig. 1b) and 6.1 Å (Fig. 1c), demonstrate the different switching modes associated with ferromagnetic coupling between layers and AFM-IEC.

When the Ir thickness is 3.5 Å in system 1 (Fig. 1b), the hysteresis shows five separate switches and therefore an AFM-IEC between each layer. The sharp switches (an abrupt change in the moment) indicate that the individual layers switch via nucleation of a small number of reversed domains at points of lower anisotropy, followed by sweeping of domain walls through the entire film. Since interpretation of microscopic switching based solely on hysteresis loops can be challenging, the conclusions about switching were confirmed by observation under a MOKE microscope. An example of one of the five switches in Fig. 1b can be seen in Supplementary Fig. S3, it confirms that the switching mechanism relies on one domain wall sweeping across the sample at each switch. This mode of switching also shows that in the AFM-IEC films there is a reduced stray field, compared to the ferromagnetically coupled films which favor maze domains, and the ferromagnetic layers switch separately. Where more rounded switches are seen while layers are switching (e.g. in Fig. 1b), this can indicate a small amount of coherent rotation, or nucleation of multiple domains before domain walls sweep across the film.

For system 1 with 6.1 Å of Ir (Fig. 1c), the hysteresis loop has a wasp-waisted shape (narrow at the center), a feature which is characteristic when maze domains (a mixture of small domains of opposite polarity, normal to the film) are present during the switching process and in the remanent state. As the switching occurs, domains with the same magnetization polarity as the applied field grow, at the expense of domains of the opposite polarity. In order for the maze domains to form, the film must be sufficiently thick to produce a large stray field, and requires that all of the layers are ferromagnetically coupled together. Thus, this wasp-waisted loop shows that the coupling between the layers is ferromagnetic for Ir spacers of 6.1 Å.

A detailed discussion of the effects of neglecting one of the spacer layers (Pt/Ir) in a system with N = 2 and N = 3, is given in the Supplementary Fig. S2.

**Figure 2:**
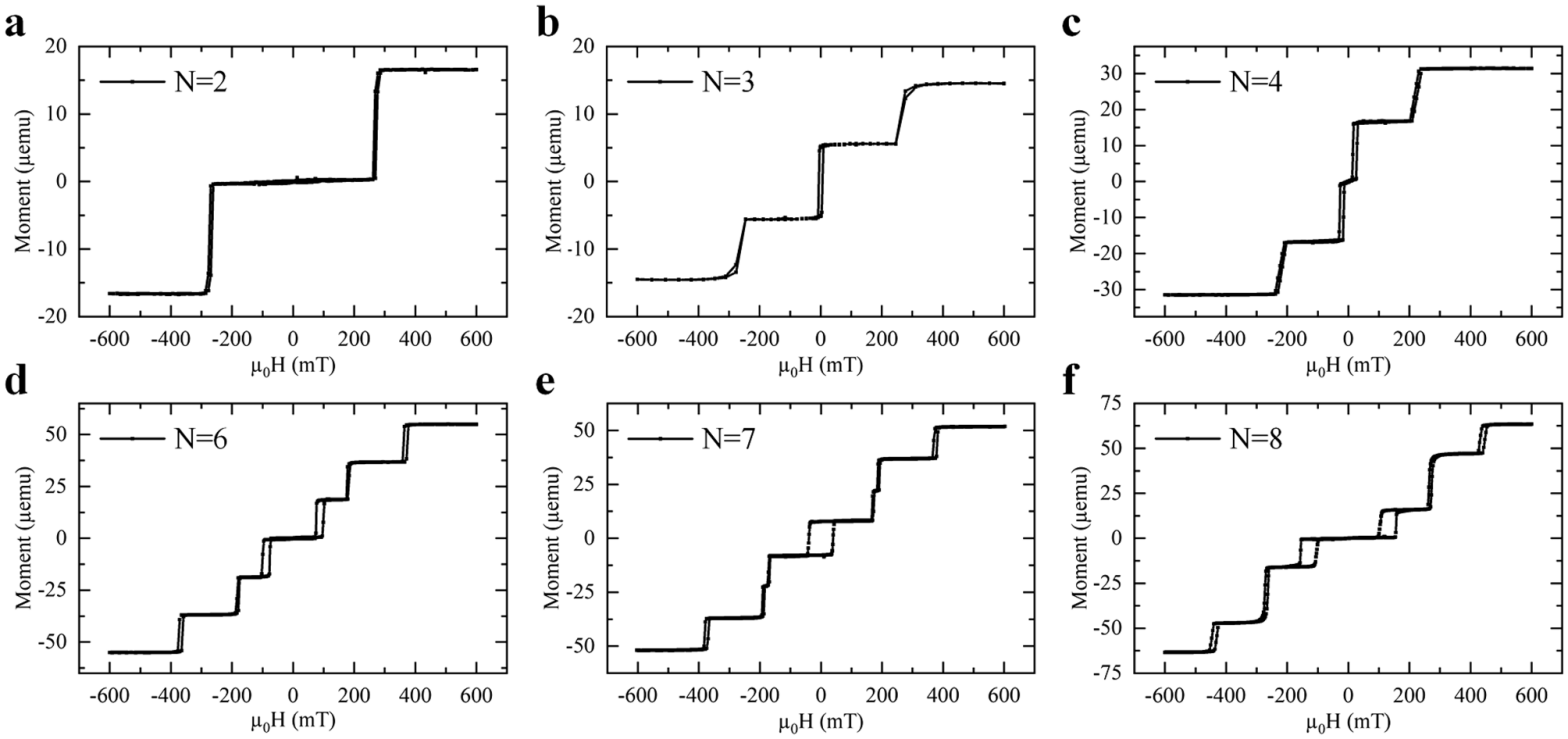
(**a**–**f**) show the hysteresis loops of system 1 performed by out-of-plane SQUID with different repetitions, varying N from 2 to 8. Up to seven repetitions, the hysteresis showed separate switches that directly correlated to the number of ferromagnetic layers. After seven repetitions, the some of the individual layers began switching together, resulting in a switch of a higher amplitude than the others i.e. of more moment.

An experimental investigation was designed to analyze system 1 as the number of repetitions increases, as shown in Fig. 2. Similar switching behaviors are observed for up to seven repetitions. We also observe that, unlike the systems with an even number of repetitions, the systems with an odd number of repetitions always had a central switch across zero. When the system has eight repetitions, we see only six switches. However, two are of double the amplitude of the other four, indicating that two layers are switching together. As this only occurs for a higher number of repetitions, this could be due to the orange-peel effect, which causes rough surfaces to couple via dipolar fields^49–51^. This investigation suggests a limit of the SAF with layer-by-layer switching i.e. the number of switches no longer correlates to the number of layers above the threshold of N = 7.

### System 2: [CoB/Ir/Pt]_x4_/CoB/Ir/Pt/[CoB/Ir/Pt]_x5_

**Figure 3:**
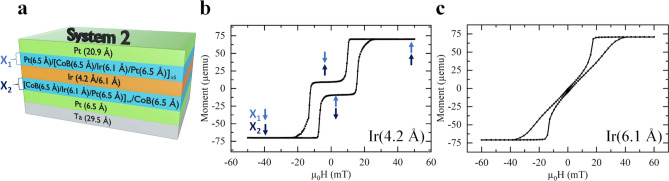
Sketch of system 2 and corresponding hysteresis measurements (**a**) Two ferromagnetic multilayers (X1 and X2) separated by 4.2 Å or 6.1 Å of Ir causing the two ferromagnetic multilayers to couple together antiferromagnetically or ferromagnetically. There is also a base of Ta and Pt, and a Pt capping layer. (**b**,**c**) Hysteresis loop performed using SQUID magnetometry with a magnetic field applied out-of-plane. (**b**) The hysteresis loop of system 2, where the blue arrows represent the total spin orientation of the two ferromagnetic multilayers (X1 and X2). Due to AFM-IEC, X_1_ and X_2_ switch separately either side of zero field, images of the sample undergoing hysteresis can be seen in Fig. 4. (**c**) The hysteresis loop of system 2 with an Ir thickness of 6.1 Å. In this case, only one switch is present and therefore ferromagnetic coupling between the ferromagnetic layers.

Figure 3a shows system 2, two multilayers of CoB, Ir and Pt, X1 and X2, which both consist of five ferromagnetic layers with positive IEC. A 4.2 Å layer of Ir, which causes a negative IEC between the two ferromagnetic multilayers, separates X1 and X2. Figure 3b and c show the SQUID-measured hysteresis loops. When the Ir thickness is 4.2 Å, it is evident that when going from a negative saturated state to a positive saturated state there are two separate switches, similarly to a SAF with only two ferromagnetic layers^32^. In our system, this SAF-like switching behavior suggests the separate switching of the two ferromagnetic multilayers, X_1_ and X_2_. The loop is open at 0 mT, indicating that it is an uncompensated SAF due to an imbalance in the material either side of the AFM coupling layer. In our case, it is due to extra moment in the top half of the system due to more CoB/Pt interfaces and induced moments in the Pt—this could be easily adjusted to create a fully compensated SAF by altering the Pt thicknesses in the bottom half of the system. The sample was grown uncompensated to aid imaging analysis of the sample.

When the thickness of the middle Ir spacer is increased to 6.1 Å, only one wasp-waisted switch occurs in the hysteresis loop (Fig. 3c), indicating that all ten CoB layers are coupled ferromagnetically. It is also noted that the hysteresis loops of the two ferromagnetic multilayers are less sharp and more wasp-waisted than the switches seen in Fig. 2a for system 1, in fact, maze domains are stabilized as discussed below.

**Figure 4:**
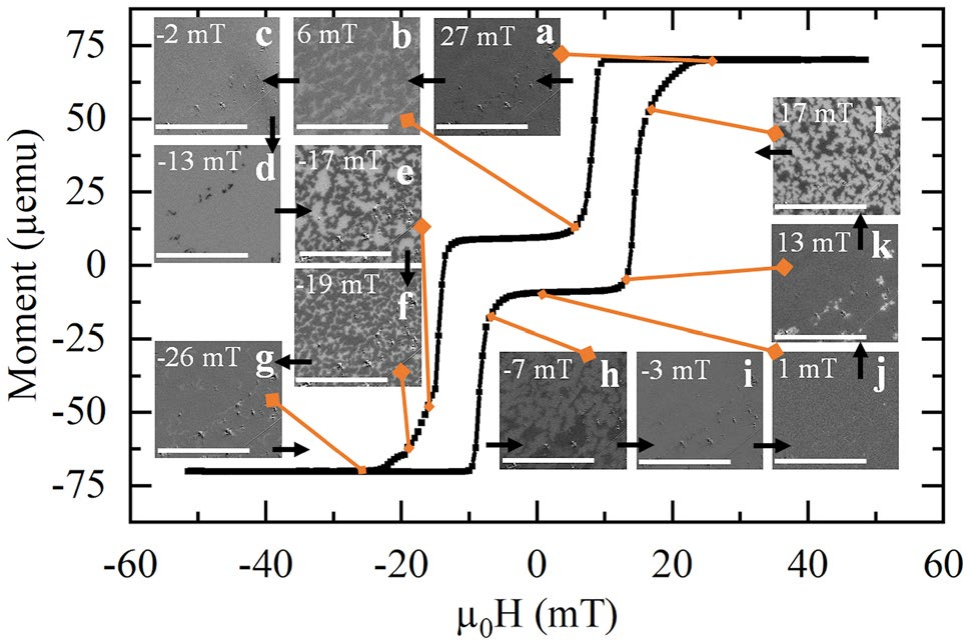
The hysteresis loop of system 2 taken via SQUID magnetometry with an out-of-plane field and images taken by Kerr microscopy with different applied out-of-plane fields during the hysteresis. Images are labelled from (**a**) to (**l**), all scale bars are 20 µm. The figure shows that when the system is taken from a positively saturated state to a negatively saturated state, it undergoes two separate switches with maze-like domains.

Figure 4 shows the same SQUID hysteresis measurement as in Fig. 3b for system 2, together with the Kerr microscopy images taken at various fields as it switches. When domains form, this appears on the image as either darker, or lighter areas depending on the orientation of the switching magnetic moments. Different contrasts are due to layers closer to the sample's surface having a clearer Kerr signal than those deeper within the sample. After starting in a saturated state at 27 mT (Fig. 4a), the field is decreased until darker, domains appear and grow, becoming dominant over the lighter domains (Fig. 4b) until they all disappear at − 2 mT (Fig. 4c). Again, dark, circular-like features of around 600 nm and other worm-like domains are observed at − 13 mT (Fig. 4d). These darker domains grow as the field is decreased further, until they become maze-like (Fig. 4e) and more dominant at − 19 mT (Fig. 4f). At the same time, the lighter domains become thinner and reduce until only small features of around 600 nm remain. All lighter domains disappear after − 26 mT (Fig. 4g). The hysteresis from a negative to positive saturated state shows the same process, with lighter domains appearing (Fig. 4h–l). Despite the limited resolution of the Kerr microscope, these images give an insight on the switching mechanism that occurs in system 2.

Therefore we were able to design an alternative type of SAF where two five-repetition multilayers experience AFM-IEC and switch separately. In addition, it shows magnetic spin textures in both ferromagnetic layers, which could be harnessed to host skyrmions.

### Effect of temperature on Systems 1 and 2

**Figure 5:**
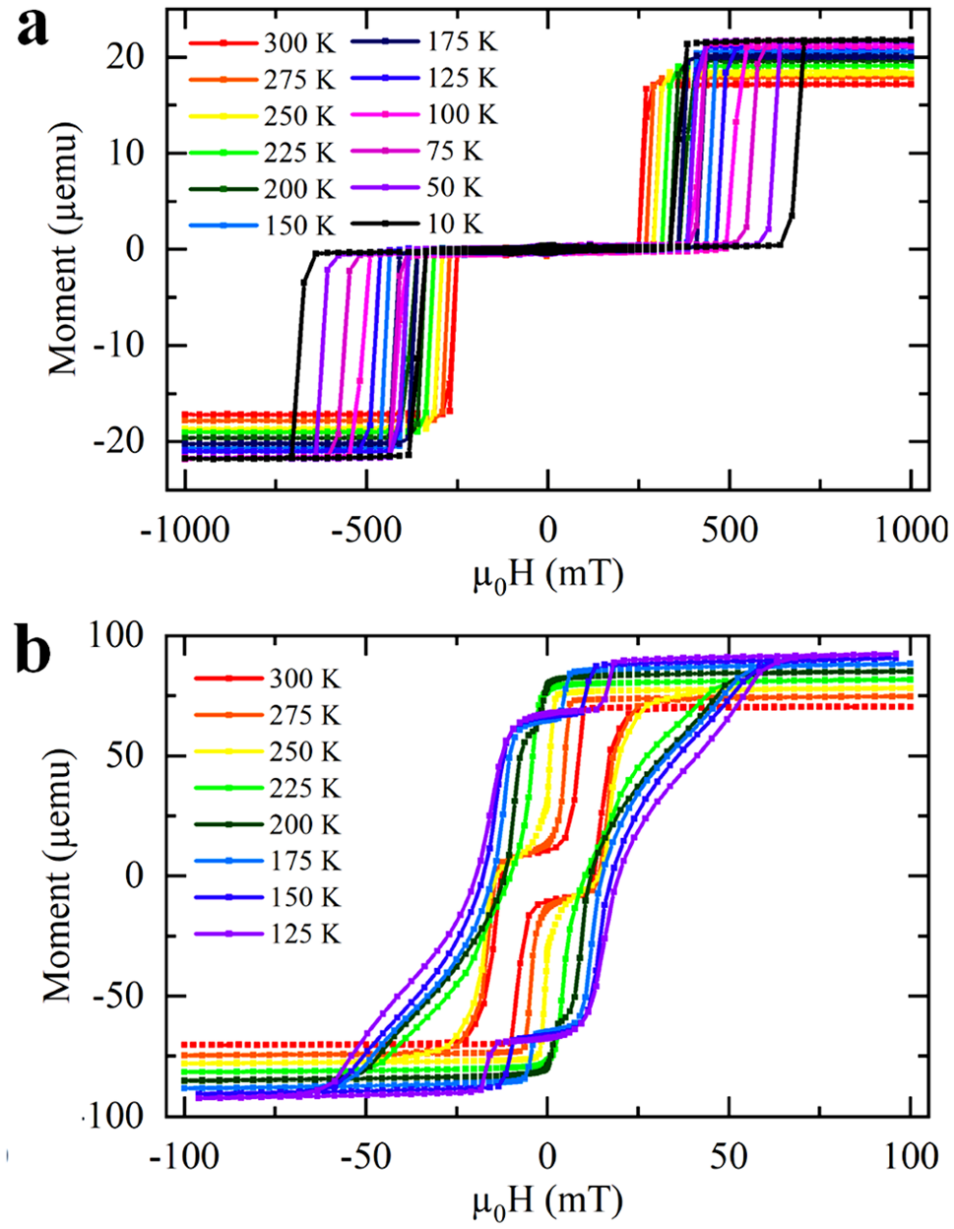
Low temperature SQUID hysteresis loops. (**a**) System 1 with two repetitions at temperatures ranging from 300 to 10 K. The Ir thickness used throughout the system was 3.5 Å, which allows the AFM-IEC. This system maintains the two switches down to 10 K, as the coercivity of the switches and the moment increases with decreasing temperature. (**b**) System 2 at temperatures ranging from 300 to 125 K. The Ir thickness used to couple together the two ferromagnetic multilayers was 4.2 Å, which allows the AFM-IEC. Unlike for system 1, below 250 K, the hysteresis loop changes, and it appears as though nearly all the ferromagnetic layers switch together.

We studied the magnetic hysteresis of systems 1 and 2 as a function of temperature. System 1 with N = 2 is found to maintain the individual layer switches down to temperatures of 10 K (see Fig. 5a), this was also true for N = 8 (Supplementary Fig. S4a). Both the coercivity of the switches and the switching field increases as the temperature decreases.

In contrast to system 1, system 2 maintains the AFM character with two switches until 250 K, where-after the hysteresis loop takes the shape of a system seemingly coupled ferromagnetically (see Fig. 5b). The coupling, coercivity and anisotropies (PMA and in-plane) all increase as the temperature is lowered, leading to changes in the hysteresis that depend on which parameter is dominant at a particular temperature.

At 225 K the shape is most likely due to both the perpendicular and demagnetizing anisotropy overcoming the AFM-IEC. Below 225 K, the hysteresis loop begins to change again showing a small switch before the main wasp-waisted section. This could be caused by the two layers in the centre directly coupled by the Ir where AFM-IEC is becoming stronger.

At 200 K, the coupling constant has not increased much, as the AFM state switch is at a similar field to the hysteresis loop at 300 K, however, the saturation field has increased substantially and the hysteresis loop appears wasp-waisted, indicating the in-plane anisotropy has increased.

At 100 K, the AFM coupling is stronger as the system switches at higher fields and remains in the AFM state for longer. The saturation field is also increasing which indicates that the demagnetizing anisotropy is still increasing.

We therefore conclude that the limit of the ordinary SAF coupling is 250 K for system 2 which is a direct consequence of the relative AFM coupling compared to the demagnetizing anisotropy. In system 2 the AFM coupling is considerably weaker than system 1 since there is only one layer spacer layer responsible for the coupling of five ferromagnetic layers.

To quantify the effect of the AFM-IEC, we computed the IEC constant for system 1 by using the following expression^52^:

1 $$J_{IEC} = - \frac{1}{2}M_{s} t_{FM} H_{sw}$$

where $M_{s}$ is the saturation magnetization, tFM is the total thickness of the ferromagnet and Hsw is the switching field, taken from the center of the loops.

Supplementary Figure S5 shows the IEC constant for system 1 with N = 2 extracted from the loops in Fig. 5a. Equation (1) does not account for hysteresis which opens in the loops, particularly at low temperatures, therefore, the calculated *J*_*IEC*_ reported in Supplementary Note 2 has more uncertainty in its values, particularly at lower temperatures, due to the difficulty of clearly identifying the switching field. Nonetheless, the temperature dependence of *J*_*IEC*_ calculated from this method has a temperature dependence comparable with that predicted for metallic spacers^36,38,39,53^, which decreases with increasing temperature^4^. This could not be estimated for system 2, using Eq. (1) as there are other factors to consider.

We ascribed this different behavior to the increase in the Co magnetic material in system 2 compared to system 1. To confirm this hypothesis, system 1 with N = 2 in Fig. 2a is grown with an increased concentration of Co. The result is shown in Supplementary Fig. S4b. We see that the increase in the Co material leads to smaller switching fields at 300 K, then, the anisotropy becomes dominant by 175 K, as in system 2.

Our results point out the high dependence of the SAF properties on the fine balance of the ferromagnetic layers thickness and compositions, as well as on the better-known spacer thickness. An increase in either the number of repetitions of the CoB layers coupled or in the Co concentration inhibits the ability of the system to follow the typical SAF behavior at low temperatures.

### Micromagnetic simulations

**Figure 6:**
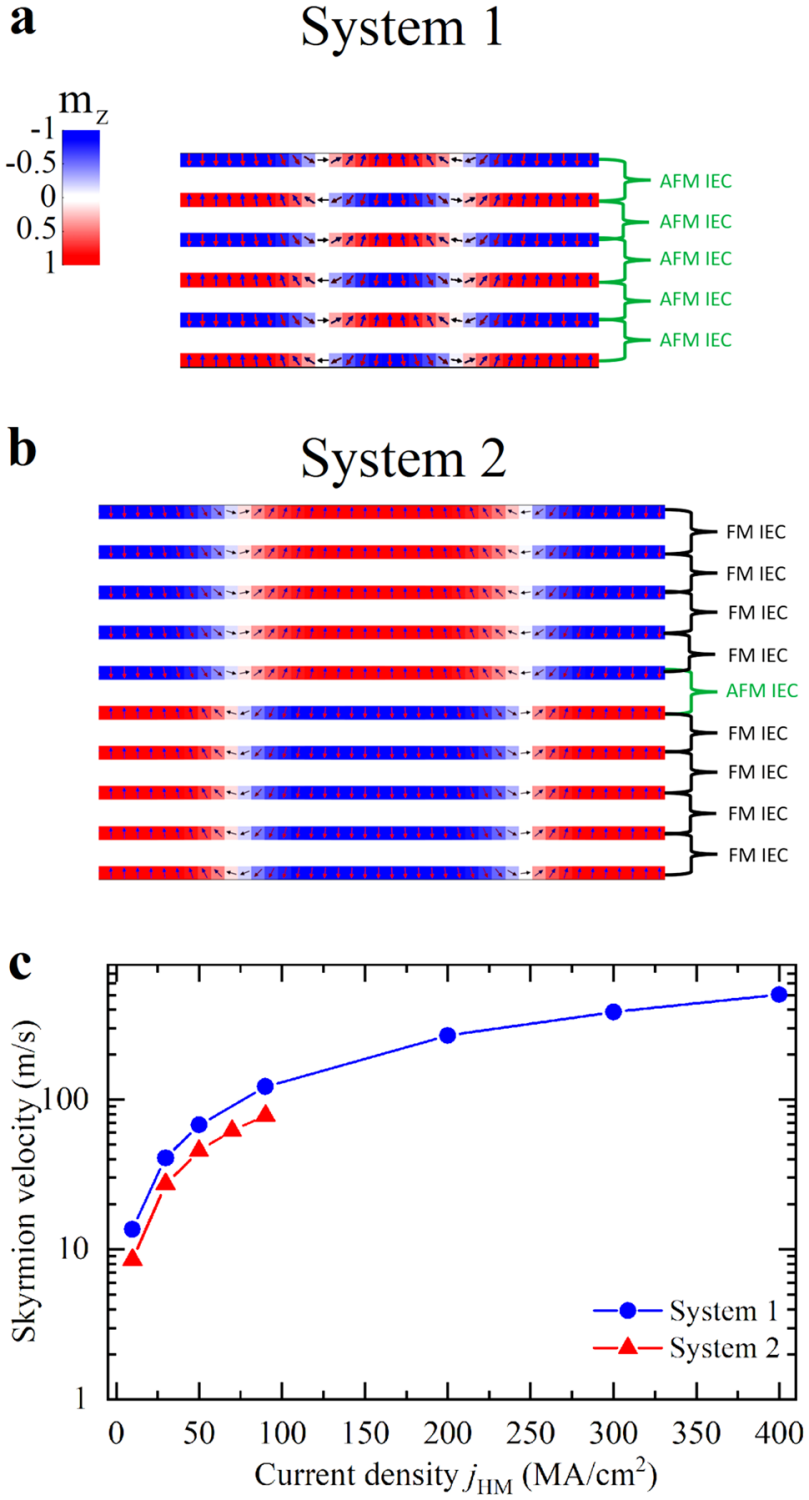
Cross section of the SAF skyrmion texture in (**a**) system 1 with N = 6 and (**b**) system 2 with the two five-repeat multilayers antiferromagnetically coupled. (**c**) Velocity-current relation for the SAF skyrmion in system 1 and 2. The velocity axis is plotted on a logarithmic scale to improve clarity in the comparison. A zero skyrmion Hall angle is observed in both cases.

We also perform micromagnetic simulations to show the potential of the experimental SAF systems considered in this work for a twofold aim to predict: the possibility of the systems to host SAF skyrmions, and the current-driven dynamics for future applications. For system 1, we consider N = 6 as in the experimental hysteresis loop shown in Fig. 2d and, for system 2, we couple two five-repetition ferromagnetic multilayers via an AFM-IEC due to the presence of the Ir layer, as in Fig. 3b. In both cases, we start the simulations from SAF skyrmions, relax the state at zero external field, and obtain a stable SAF Néel skyrmion with a diameter of about 50 nm in system 1 (Fig. 6a), and 90 nm in system 2 (Fig. 6b).

To study the skyrmion dynamics driven by electrical current, we apply the current throughout the Pt layers which is able to excite a damping-like SOT via the spin Hall effect (SHE). The experimental Fig. 2d shows a compensated SAF, therefore we expect the dynamics in system 1 to be similar to previously reported SAF results^16,20^. Indeed, Fig. 6c shows high skyrmion velocities with zero skyrmion Hall angle.

Differently from system 1, system 2 exhibits an uncompensated nature. Nonetheless, our micromagnetic simulations still show the absence of the skyrmion Hall angle, where the skyrmion moves only in the direction of the current with a velocity-current relation as shown in Fig. 6c. However, the achieved velocities are not as large as the previous SAF system and studies on ideal bilayer SAFs^16,20^. We ascribe this difference to the fact that the AFM coupling acts only between the central layers. It is well-known that a larger AFM coupling allows for the application of a larger SOT^12^. In system 2, the majority of the layers are coupled ferromagnetically and this reduces the overall beneficial effect of the AFM coupling, thus limiting the maximum applicable SOT, and therefore the applicable current density. Beyond this value, the skyrmion undergoes a lateral expansion^19,54^, which elongates the skyrmion texture in the direction perpendicular to the motion.

The different dynamical properties of skyrmions in the two systems suggests different applications of the two types of SAF. System 1 could be more suitable for memory, whereas system 2 could be more useful for unconventional applications.

## Summary and conclusions

We have investigated a SAF platform based on CoB/Ir/Pt multilayers with the aim to exploit its potential benefits for future domain wall and skyrmion based applications including in neuromorphic computing^55,56^, stochastic computing^57^, reservoir computing^56,58–60^ and dynamical neurons^61^.

We have focused on two systems, *system 1* and *system 2.* The key results are: the stability of the SAF (system 1) up to seven repetitions, and the limitations with temperature of the SAF when more magnetic material is coupled together.

As a function of temperature, system 1 maintained the SAF behavior down to 10 K when N = 2 and N = 8. System 2 showed a different behavior below 250 K, defining the limits of where the AFM-IEC is the dominant effect. This trend was repeated when system 1 with N = 2 was made with an increased cobalt to boron ratio, indicating the dependence of the IEC on the magnetic material coupled. The pros of system 1 are the strong AFM-IEC, which remains robust to both increasing the number of repetitions and temperature. The con is that it is not simple to nucleate skyrmions. On the other hand, in system 2, it is easier to create domain structures and field-tunable spin patterns, however the coupling is weaker and more sensitive to temperature variations. Theoretical predictions show that for system 1, high skyrmion velocities are expected compared to system 2. These results anticipate that system 2 can be very useful for unconventional applications of skyrmion devices where thermal effects and excitation of skyrmion modes can play a key role^57^. On the other hand, system 1 can be used for more standard applications, such as magnetic storage, where the skyrmions should move faster and can be subjected to larger currents without the skyrmion Hall effect. Finally, our results distinguish the limits and capabilities of PMA SAF systems for future development of high performance racetracks, both for skyrmions and domain walls.

## Methods

### Experimental

The systems were grown via DC magnetron sputtering at a base pressure of ~ 10^−9^ mbar and an argon pressure of 4.6 × 10^−3^ mbar. For Ta, Pt, Ir, and CoB sequentially, the growth power densities were 4.08 W/cm^2^, 1.53 W/cm^2^, 1.53 W/cm^2^, and 2.55 W/cm^2^ and the growth rates were 0.9 Å/s, 0.72 Å/s, 0.4 Å/s, and 0.24 Å/s. All films were deposited on thermally oxidized silicon at room temperature. For all samples, a base layer of Ta was used as a buffer and Pt was used to cap the system to prevent oxidation. The systems were characterized using low angle X-ray scans to determine the thickness of the different layers. This was important as a small difference in the thickness could change the nature of the system significantly. The scans were fitted using GenX^48^ to calculate the thicknesses of the materials (see Supplementary Fig. S1 and Supplementary Table S1).

Measurements were done using SQUID-VSM (vibrating sample magnetometry) to obtain the hysteresis loops and the area was determined by imaging the sample with a scale and calculating the mm to pixel ratio. The imaging technique used in this study was Kerr microscopy in polar mode. To obtain the images, the following procedure was undertaken; the system is first saturated in a high magnetic field, then the field is removed, and a background image is taken in a uniform state. This image was subtracted from the displayed images in order to get a better contrast of the domains.

### Micromagnetic model

PETASPIN, a state-of-the-art micromagnetic solver, was used to perform the micromagnetic simulations. The solver numerically integrates the Landau-Lifshitz-Gilbert (LLG) equation by applying the time solver scheme Adams–Bashforth^62,63^:

2 $$\frac{{d{\mathbf{m}}}}{d\tau } = - \left( {{\mathbf{m}} \times {\mathbf{h}}_{{{\mathbf{eff}}}} } \right) + \alpha_{G} \left( {{\mathbf{m}} \times \frac{{d{\mathbf{m}}}}{d\tau }} \right)$$

where **m** = **M**/*M*_*S*_ is the normalized magnetization, *α*_*G*_ is the Gilbert damping, and *τ* = *γ*_0_ *M*_*S*_ *t* is the dimensionless time, which uses *γ*_0_ the gyromagnetic ratio and *M*_*S*_ the saturation magnetization. The normalized effective magnetic field, **h**_**eff**_, includes the exchange, interfacial DMI, magnetostatic, anisotropy and external fields^64,65^. The effective field also includes the interlayer exchange coupling ${\mathbf{h}}_{ex,i}^{inter} = \frac{{J_{IEC} }}{{\mu_{0} M_{s}^{{j^{2} }} t_{{{\text{NM}}}} }}{\mathbf{m}}^{j}$ where *i,j* are the indices of two consecutive ferromagnetic layers along the z-axis, i.e. sample thickness. This equation uses the interlayer exchange coupling constant *J*_*IEC*_, the vacuum permeability* µ*_0_, and the thickness of the non-magnetic layer* t*_NM_.

We simulate the magnetic multilayer system 1 in Fig. 2d [CoB/Ir/Pt]_xN_ with N = 6 repetitions of a 6.5 Å thick CoB ferromagnet separated by a 13 Å thick Ir/Pt non-magnetic layer. This system is characterized by a negative AFM-IEC constant *J*_*IEC*_ between consecutive CoB layers. System 2 in Fig. 3b [CoB/Ir/Pt]_x5_/Ir/[CoB/Ir/Pt]_x5_ is simulated by ten repetitions of a 6.5 Å thick CoB ferromagnet separated by a 13 Å thick Ir/Pt non-magnetic layer. The Ir layer separating the two five-repetition multilayers is fixed to 6.5 Å. In addition, system 2 is characterized by different IEC constants *J*_*IEC*_ between neighboring CoB layers: one within each [CoB/Ir/Pt]_x5_ multilayer, which is due to the Pt(6.5 Å)/Ir(6.1 Å), and has a positive value of about 1 mJ/m^2^^30^ thus promoting ferromagnetic coupling. Plus, a negative value between the two [CoB/Ir/Pt]_x5_ multilayers due to the middle Ir layer of 4.2 Å, which promotes the antiferromagnetic alignment of the two multilayers. According to Ref.^30^, the value is smaller than that in the ferromagnetic case. In order to simulate this system, we implemented a variable IEC into our micromagnetic solver, PETASPIN.

For the CoB, we used the following parameters: saturation magnetization *M*_s_ = 800 kA/m similar to the experimental value of 720 kA/m, uniaxial perpendicular anisotropy constant *K*_u_ = 0.57 MJ/m^3^, exchange constant *A* = 10 pJ/m, and interfacial DMI constant *D* = 1.5 mJ/m^2^ similar to Ref.^66^. All the simulations are carried out at zero external field. We use a discretization cell size of 4 × 4 × 0.65 nm^3^ and simulate the two systems with in-plane dimensions of 800 × 800 nm^2^.

The first set of simulations are performed to determine the minimum value of the AFM-IEC constant allowing for a stable SAF. We start the simulations with a uniform ferromagnetic initial state and increase the AFM-IEC to the value at which this initial ferromagnetic state is no longer stable. For *J*_*IEC*_ ≤ − 0.25 mJ/m^2^, only a SAF state is stable. Therefore, we use this value for both system 1 and 2 when an AFM-IEC is considered.

When considering the SOT driven skyrmion dynamics, a damping-like torque is included, as in Eq. (3):

3 $$- \frac{{g\mu_{B} \theta_{SH} }}{{2\gamma_{0} eM_{S}^{2} t_{FM} }}\left[ {{\mathbf{m}} \times \left( {{\mathbf{m}} \times \left( {\hat{z} \times {\mathbf{j}}_{{{\mathbf{HM}}}} } \right)} \right)} \right]$$

where *g* is the Landé factor, *µ*_*B*_ is the Bohr magneton, *θ*_*SH*_ is the spin-Hall angle which is set to 0.1, *e* is the electron charge. The unit vector along the out-of-plane direction is described by $\hat{z}$, and the electrical current density flowing into the Pt/Ir non-magnetic layers (we assume that flows entirely into the Pt) allows for the SOT from the SHE. Particularly, it has been demonstrated via experimental measurements that in this type of multilayer, a damping-like torque arises^66^.

### Supplementary Information

Supplementary Information.

## Data Availability

The datasets generated during and/or analyzed during the current study are available from the corresponding author upon reasonable request.
