# Supplementary material for: Antiferromagnetic interlayer exchange coupled Co68B32/Ir/Pt multilayers
Source: Sci Rep. 2024 Jan 2;14:95. doi: 10.1038/s41598-023-49976-4 (PMC10761723; doi:10.1038/s41598-023-49976-4)
Supplement: Supplementary file 1 — Supplementary Information. [file 41598_2023_49976_MOESM1_ESM.docx]

# SUPPLEMENTARY INFORMATION

# Antiferromagnetic interlayer exchange coupled Co_68_B_32_/Ir/Pt multilayers

Emily Darwin^1,2^, Riccardo Tomasello^2^, Philippa M. Shepley^1^, Nathan Satchell^1#^, Mario Carpentieri^2^, Giovanni Finocchio^3^* and B J Hickey^1^*

^1^School of Physics and Astronomy, University of Leeds, Leeds LS2 9JT, UK.

^2^Department of Electrical and Information Engineering, Politecnico di Bari, Via E. Orabona 4, 70125 Bari, Italy

^3^Department of Mathematical and Computer Sciences, Physical Sciences and Earth Sciences, University of Messina, Messina 98166, Italy.

^#^Now at the Department of Physics, Texas State University, San Marcos, Texas 78666 U.S.A.

*[gfinocchio@unime.it](mailto:gfinocchio@unime.it)

*[b.j.hickey@leeds.ac.uk](mailto:b.j.hickey@leeds.ac.uk)

**
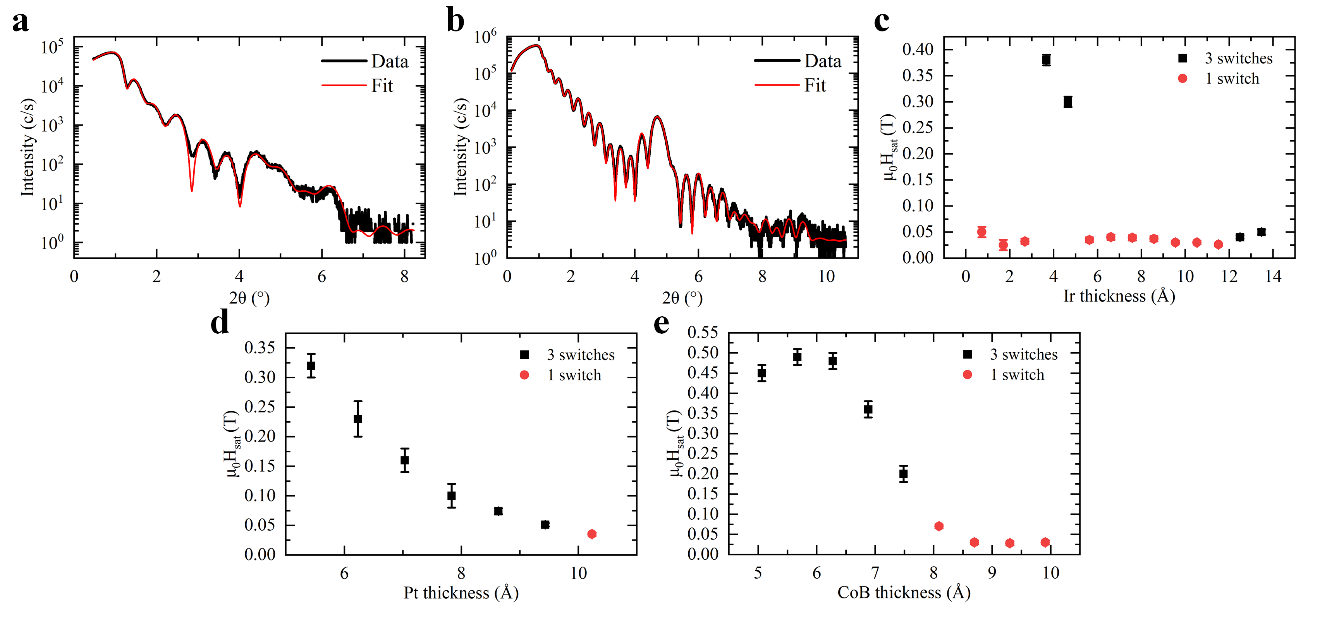
Supplementary Note 1 – Material characterization**

**Supplementary Figure S1.** X-ray reflectivity scan for (**a**) System 1 with three repetitions and (**b**) System 2, plotted against 2θ. The red lines indicate the fit calculated using GenX^1^, from which the thicknesses were obtained. (**c**) – (**e**) System 1 with three repetitions was grown as a function of Ir/Pt/CoB thickness and plotted against the saturation field taken from out-of-plane SQUID measurements. Small variations in the set thicknesses from system 1 in Fig. 1a are stated for each figure. There are either three switches during the hysteresis loop, and therefore antiferromagnetic interlayer exchange coupling (AFM-IEC) between each ferromagnetic layer, or only one switch and therefore ferromagnetic IEC. (**c**) It is observed that the AFM-IEC occurs at Ir thicknesses of 3.5 Å - 5 Å, and 12.5 Å - 14 Å and the coupling strength of Ir after 12 Å, is lower than around 4 Å as expected^2^. In this figure, the CoB is 6.9 Å and the Pt is 5.8 Å. (**d**) AFM-IEC for Pt thicknesses between 5 and 10 Å. Below this, the system does not exhibit PMA and above 10 Å ferromagnetic coupling (one switch) is observed. The coupling strength decreases with increasing Pt thickness. In this figure, the Ir is 4.1 Å and the CoB is 6.5 Å. (**e**) AFM-IEC for CoB thicknesses of between 5 and 8 Å. Below this, the system does not exhibit PMA and above 8 Å ferromagnetic coupling (one switch) is observed. The coupling strength peaks at around 5.5 Å, then decreases with increasing CoB thickness. In this figure, the Ir is 4.3 Å and the Pt is 5.6 Å.

The antiferromagnetic coupling is still present with a Pt layer between the Ir and CoB, however, its strength is reduced^3,4^. System 1 with N=3 was investigated as a function of Pt and CoB thickness. In Fig. S1c – S1e, we plot the saturation field to represent the coupling and not the IEC, as there was a combination of ferromagnetic and antiferromagnetic coupling. The saturation field was taken from the hysteresis loops measured with an out-of-plane field via SQUID magnetometry. For Pt thicknesses (Fig. S1d) between 5 and 10 Å, AFM-IEC was present as three switches, one for each CoB layer, are observed. Below this, the system did not exhibit PMA and above 10 Å, ferromagnetic coupling and therefore one switch is observed. As the thickness of the Pt layer is increased, the saturation field decreases, suggesting the coupling strength reduces. Fig. S1e shows AFM-IEC for CoB thicknesses of between 5 and 8 Å. Similarly to Pt, below 5 Å, the system does not exhibit PMA and above 8 Å ferromagnetic coupling is observed. The coupling strength reaches a maximum at around 5.5 Å, then it decreases as the CoB thickness increases.

|  | Thickness (Å) | Roughness (Å) | Density Difference |
| --- | --- | --- | --- |
| Cap Pt | 20.9 ± 0.2 | 2.88 ± 0.06 | 99% ± 5% |
| Middle Ir | 4.2 ± 0.2 | 1.27 ± 0.2 | 96.3% ± 0.6% |
| Ir | 6.11 ± 0.09 | 1.0 ± 1.0 | 96.3% ± 0.69% |
| CoB | 6.50 ± 0.02 | 5.0 ± 0.1 | 105% ± 10% |
| Pt | 6.48 ± 0.09 | 5.06 ± 0.3 | 102% ± 6% |
| Base Ta | 29.5 ± 0.3 | 3.2 ± 0.3 | 93% ± 6% |

**Supplementary Table S1.** Table displaying the values taken from the GenX fit of system 2, shown in Fig. S1b. The layer thicknesses, roughness, and density as a percentage of literature density values.

**Supplementary Note 2 – Hysteresis measurements
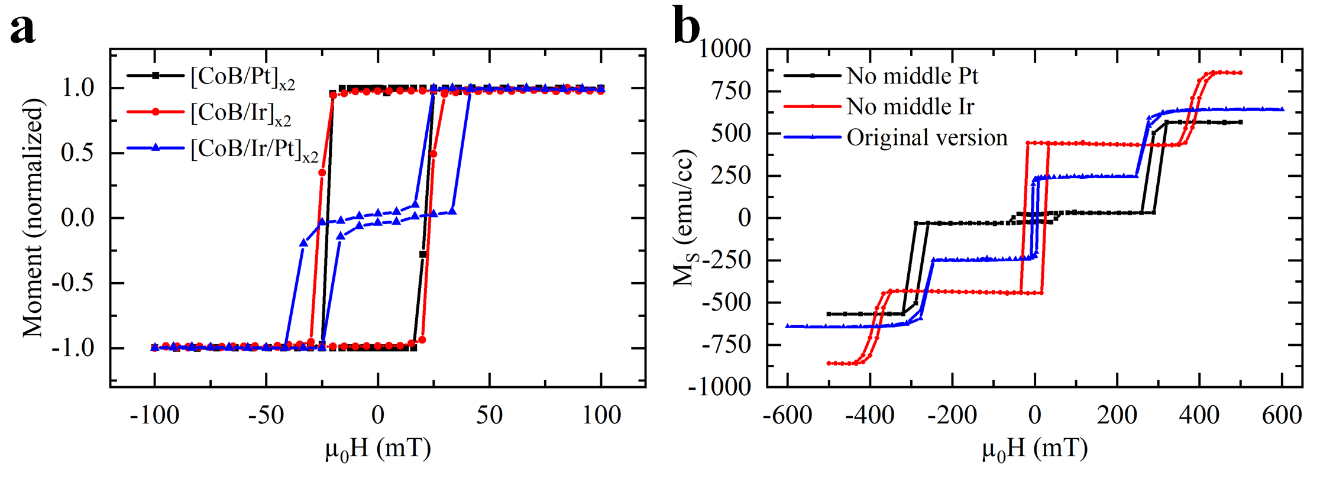
**

**Supplementary Figure S2.** Hysteresis loops for (**a**) System 1 with two repetitions, compared with the system when the Ir or Pt is removed from the multilayer. The original version of system 1, with both Pt and Ir in the spacer, has the expected two switches. Both without Ir and without Pt in the multilayer, the hysteresis loop no longer shows AFM-IEC. This was measured via laser MOKE, hence the moment is normalized. (**b**) System 1 with three repetitions. The original version of system 1, with both Pt and Ir in the spacers, has the expected three switches. This is compared to the system grown without either the middle Ir layer or middle Pt layer. Three switches are observed in both cases, however, both amplitudes and coercivities of the individual switches vary. A base of Ta(30 Å)/Pt(30 Å), and a cap of Pt(30 Å) were always present. The saturation magnetization (M_S_) was measured via SQUID.

To achieve a better understanding of the switching mechanisms, system 1 with N=2 was grown without either the Pt or Ir in the multilayer, shown in Fig. S2a. The nominal thicknesses are CoB = 6 Å, Ir = 4 Å, and Pt = 6 Å. As expected, without the Ir present, the system no longer shows the AFM-IEC. There is still PMA, however, ferromagnetic IEC is observed, i.e. one switch only. When the Ir is present but the Pt is not, the system's hysteresis is very similar to that of the system with the Ir absent. This means that the Pt plays a vital role within the system, likely due to the induced moments in the Pt supporting the CoB magnetization.

To further explore the role of the Pt, system 1 with three repetitions was grown without either the middle layer of Ir or middle layer of Pt and compared to the full multilayer system, as shown in Fig. S2b. The thicknesses were measured via GenX, the 'Original version' sample thicknesses are CoB = 6.6 Å, Ir = 4.4 Å, and Pt = 5.6 Å. The 'No middle Pt' sample thicknesses are CoB = 5.8 Å, Ir = 4.1 Å, and Pt = 5.6 Å and the 'No middle Ir' sample thicknesses are CoB = 6.6 Å, Ir = 4.1 Å, and Pt = 6.0 Å. The results show that the system without an Ir layer still has three switches for each CoB layer. When considering how the spins change orientation, one possibility for how the spin flips could occur with two layers coupled ferromagnetically and two layers coupling antiferromagnetically is as follows: ↑↑↑, ↓↑↑, ↓↓↑, ↓↓↓. In this case, there would still be three individual switches observed. The outer two switches represent the switching of the bottom CoB layer, which is coupled antiferromagnetically to the two upper layers of CoB which, without the Ir between them, couple ferromagnetically. The increased amplitude of the middle switch indicates that the two CoB layers coupled together ferromagnetically possess more moment, this may be due to the Pt now having two interfaces with CoB instead of one. The outer switches also have a slightly higher switching field. The system without Pt shows similar outer switching fields, however, with a larger coercivity and amplitude. The middle switch is very different, with a very low amplitude and larger coercivity. This could be due to the considerable deduction in moment with a missing CoB/Pt interface.

These results show the necessity of each component of the system to create a stable SAF. If even one layer of the Pt is removed, the system would not have the same SAF characteristics, partially due to the polarization of the Pt at the ferromagnetic interface. Without the Ir the SAF cannot be created, however, by removing only one layer of Ir in an N=3 system, we still observe SAF behavior, with some differences from the original system 1, as two CoB layers switch together.


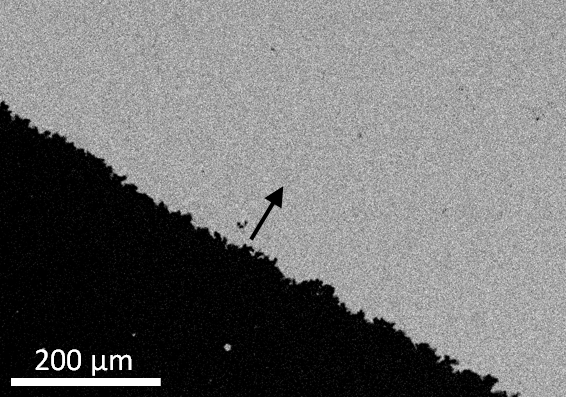


**Supplementary Figure S3.** This shows the Kerr microscopy image of a darker domain wall sweeping across system 1 with N=5 during hysteresis at an out-of-plane magnetic field of 257 mT. The arrow represents the direction of the domain wall motion.

**Supplementary Note 3 – Temperature dependence**


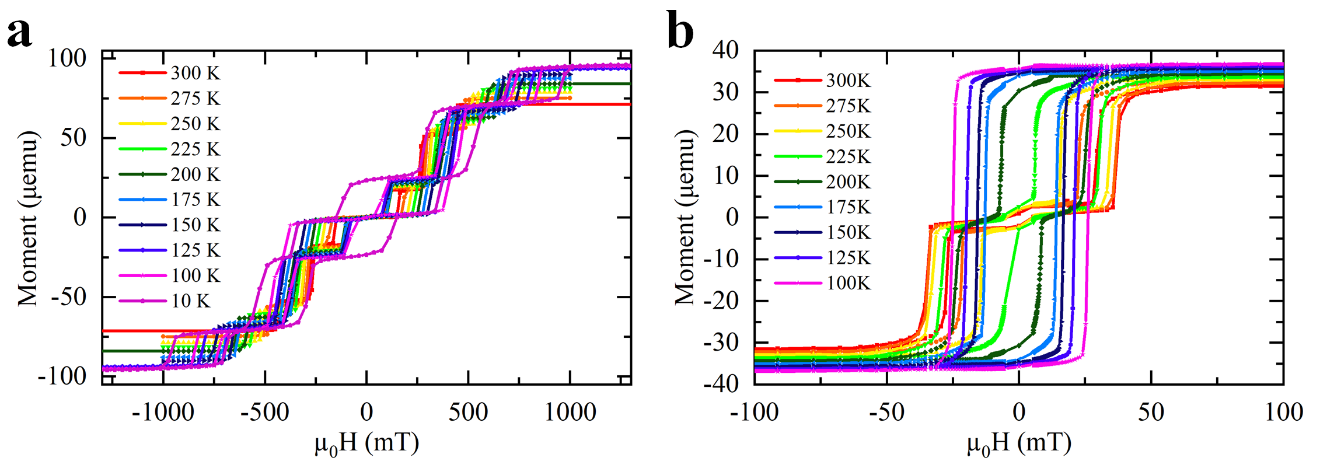


**Supplementary Figure S4.** SQUID magnetometry measurements to low temperatures. (**a**) The hysteresis loops for system 1 for N=8, between 300 K and 10 K. This system maintains the separate down to 10 K, as the coercivity of the switches increase and the moment increases with decreasing temperature. (**b**) Data from system 1 of thicknesses: [CoB(6.5 Å)/Ir(4 Å)/Pt(6.3 Å)]_x2_, however, the ratio of cobalt to boron is higher than all other samples due to co-sputtering Co at the same time as the CoB. Unlike for system 1 with two repetitions without co-sputtered Co, below 200 K, the hysteresis loop changes, and it appears as though nearly all the ferromagnetic layers switch together.

Equation S1 was used to confirm the relationship of the interlayer exchange coupling, *J_IEC_* with temperature, *T*. *T_0_ = ħν_F_/2πk_B_L*, when *ν_F_* is the Fermi velocity and *L* is spacer thickness^5^.

$J_{IEC}(T)=J_{0}\frac{\frac{T}{T_{0}}}{\sinh\left( \frac{T}{T_{0}} \right)}$ (S1)

**
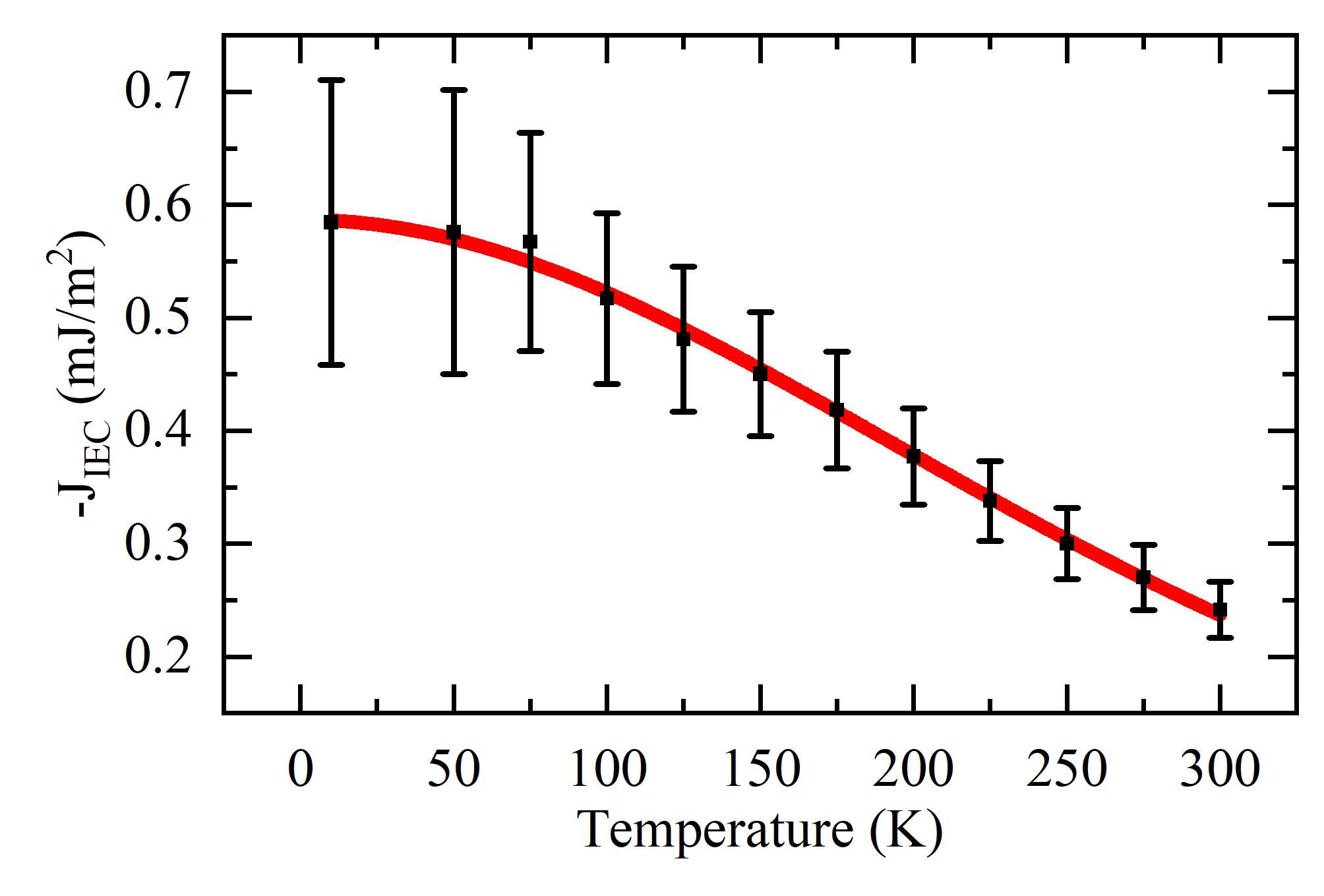
**

**Supplementary Figure S5.** The interlayer exchange coupling constant plotted against temperature for the system 1 with N=2, fitted with Equation S1, shown by the red line. The error bars become larger at low temperatures due to the hysteresis loops opening at lower temperatures, and therefore a larger range of H_sw_. The temperature dependence is comparable with that predicted for metal spacers.

**References**

1. Björck, M. & Andersson, G. GenX : an extensible X-ray reflectivity refinement program utilizing differential evolution. *J. Appl. Crystallogr.* **40**, 1174–1178 (2007).

2. Parkin, S. S. P. Systematic variation of the strength and oscillation period of indirect magnetic exchange coupling through the 3 d , 4 d , and 5 d transition metals. *Phys. Rev. Lett.* **67**, 3598–3601 (1991).

3. Karayev, S. *et al.* Interlayer exchange coupling in Pt/Co/Ru and Pt/Co/Ir superlattices. *Phys. Rev. Mater.* **3**, 3-9 (2019).

4. Legrand, W. *et al.* Room-temperature stabilization of antiferromagnetic skyrmions in synthetic antiferromagnets. *Nat. Mater.* **19**, 34–42 (2020).

5. Bruno, P. Theory of interlayer magnetic coupling. *Phys. Rev. B* **52**, 411–439 (1995).
